# Consciously Feeling the Pain of Others Reflects Atypical Functional Connectivity between the Pain Matrix and Frontal-Parietal Regions

**DOI:** 10.3389/fnhum.2017.00507

**Published:** 2017-10-20

**Authors:** Thomas Grice-Jackson, Hugo D. Critchley, Michael J. Banissy, Jamie Ward

**Affiliations:** ^1^School of Psychology, University of Sussex, Falmer, United Kingdom; ^2^Sackler Centre for Consciousness Science, University of Sussex, Falmer, United Kingdom; ^3^Brighton and Sussex Medical School, University of Sussex, Falmer, United Kingdom; ^4^Department of Psychology, Goldsmith's College, University of London, London, United Kingdom

**Keywords:** social neuroscience, empathy, empathy for pain, vicarious pain, shared representations, rTPJ

## Abstract

Around a quarter of the population report “mirror pain” experiences in which bodily sensations of pain are elicited in response to viewing another person in pain. We have shown that this population of responders further fractionates into two distinct subsets (Sensory/localized and Affective/General), which presents an important opportunity to investigate the neural underpinnings of individual differences in empathic responses. Our study uses fMRI to determine how regions involved in the perception of pain interact with regions implicated in empathic regulation in these two groups, relative to controls. When observing pain in others (minor injuries to the hands and feet), the two responder groups show activation in both the sensory/discriminative and affective/motivational components of the pain matrix. The control group only showed activation in the latter. The two responder groups showed clear differences in functional connectivity. Notably, Sensory/Localized responders manifest significant coupling between the right temporo-parietal junction (rTPJ) and bilateral anterior insula. We conclude that conscious experiences of vicarious pain is supported by specific patterns of functional connectivity between pain-related and regulatory regions, and not merely increased activity within the pain matrix itself.

## Introduction

For some people, seeing another person in pain, such as having an injection or falling off a bicycle, results in reportable pain-like experiences. These people have been referred to as mirror-sensory or mirror-pain synaesthetes (Fitzgibbon et al., 2012b) or as pain responders (Osborn and Derbyshire, 2010). Our recent study found a prevalence rate of mirror-pain of 27%, using a large scale screening questionnaire (*n* = 500+) and a k-means cluster analysis to classify participants (Grice-Jackson et al., 2017). This has the advantage of being a data driven approach such that groups are determined based on the similarity of their vicarious pain experiences rather than arbitrary cut-offs imposed by an experimenter. Grice-Jackson et al. (2017) found two sub-groups of mirror-pain responders with qualitatively distinct vicarious pain experiences characterized by differences in standardized descriptors of physical pain (Melzack, 1987), and the extent to which the evoked pain was localized. One group that we termed Sensory/Localized responders (S/L) reported sensory descriptors (e.g., sharpness) that were localized to a specific region of the body (typically the same location as the observed pain), and a second group that we termed Affective/General (A/G) responders reported affective descriptors (e.g., nauseating) that were generalized to the whole body. The validity of these groupings was established by showing that the groups dissociate on other measures. When observing pain, the Sensory/Localized responders, showed significant differences on a measure of neural synchrony (electroencephalography/EEG suppression of mu and beta rhythms) that has previously been linked to somatosensory processing (e.g., Ritter et al., 2009). This pattern was not present in controls or the Affective-General responders (Grice-Jackson et al., 2017), raising the possibility that previous results in the literature were driven by some individuals rather than reflecting a population-level trait. In terms of brain structure, using voxel-based morphometry (VBM), the two responder groups could be reliably distinguished from controls but had a similar profile to each other; namely, increased gray matter in somatosensory cortex and anterior insula, reduced gray matter in right temporo-parietal junction, rTPJ (Grice-Jackson et al., 2017).

These earlier observations provide initial evidence that individual differences in the phenomenological characteristics of vicarious pain are meaningful and underpinned by systematic differences in brain structure and function. However, our knowledge of the underlying brain systems is limited. The only previous fMRI study of mirror pain classified participants according to whether they had one or more localized pain responses when observing a set of videos/images of pain (Osborn and Derbyshire, 2010). In this study, pain responders were reported to have greater activity when observing pain, relative to control participants, in regions including anterior insula and secondary somatosensory cortex. Here, we aimed to extend this finding in two important ways. Firstly, we sought to characterize differences between our recently discovered subtypes of mirror pain. Secondly, we aimed to investigate the distinct underlying functional connectivity between brain regions supporting these types of vicarious pain response.

The central processing of pain takes places in a series of interconnected neural regions known collectively as the pain matrix (Melzack, 1999). However, it should be noted that regions involved in the perception of pain typically process other kinds of related information too (see Iannetti and Mouraux, 2010). The pain matrix is often parcellated into two conceptually different subdivisions known as the affective-motivation subdivision (processing the affective qualities of emotion preparedness of pain) and the sensory-discriminative subdivision (which processes the sensory aspects of pain) (Peyron et al., 2000). Correspondingly, we predicted that our Sensory/Localized and Affective/General responders will differentially activate these sub-systems. Moreover, within the Sensory/Localized group, we further anticipated a greater somatotopic response to observed pain whereby viewed pain to the hand or foot activates hand or foot regions of somatosensory cortex respectively. In normative populations (i.e., that do not separate out the presence/absence of mirror pain), there is consistent activation in the affective/motivation regions of the pain matrix (notably mid-cingulate cortex and anterior insula) when observing others in pain (Lamm et al., 2011). This occurs also when pain is implied, but not directly observed. It is argued that sensory/discriminative regions of the pain matrix may only be activated when the site of injury is observed, and not when pain is merely implied (e.g., via facial grimace or a symbolic cue; Lamm et al., 2011). Brain stimulation studies also suggest the “sensory simulation” of the pain of others when directly observing pain (Avenanti et al., 2005; Bufalari et al., 2007). However, these studies did not take into account the contribution of individual differences in vicarious experience. EEG has revealed a greater modulation of somatosensory evoked responses when viewing pain in mirror-pain responders compared to controls (Fitzgibbon et al., 2012a). This observation suggests that mirror pain is linked to differences in neural processing at the level of cortical sensory processing, rather than being merely an enhanced affective response.

Most models of empathy for pain assume not only activity in shared representations of pain (whether affective and/or sensory) but also regions outside of the pain matrix that are involved in selectively orienting toward self/other either in terms of bodily location (perspective taking) or in terms of orienting toward salient social and personal characteristics such as race (Decety and Jackson, 2006; Decety, 2011; Bird and Viding, 2014). For example, in a neurotypical sample, training the ability to regulate self-other representations has been linked with changes in the degree of sensory simulation of the pain of others when directly observing pain (de Guzman et al., 2016). These control mechanisms are needed to dynamically modulate the focus of attention toward other people (and suppress one's own feelings) or, conversely, to be able to focus on one's own feelings and suppress that of others (i.e., the down-regulation of empathy). One region that has been implicated as acting as a switch between self and other is the right temporoparietal junction, TPJ (Bird and Viding, 2014; Lamm et al., 2016). According to Ward and Banissy (2015) a disruption of this rTPJ mechanism in mirror pain (and mirror touch synaesthesia) underlies the tendency to experience the pain of others. In effect, for these individuals, pain is more likely to be shared rather than selectively attributed to self or other. Evidence for a role of this region in mirror pain comes from structural brain imaging studies where reduced rTPJ gray matter density is observed in people with both Sensory/Localized and General/Affective mirror pain (Grice-Jackson et al., 2017), and in people with the related symptom of mirror-touch synaesthesia (Holle et al., 2013). The present study considers in more detail the role of this region in our fMRI study of vicarious pain.

In summary, our hypothesis is that mirror-pain phenomenology is linked to increased activity of regions implicated in physical pain when observing pain. More specifically, we predict that Sensory/Localized responders will have increased activity in the sensory-discriminative sub-division of the pain matrix, whereas Affective/General responders will have increased activity in the affective-motivational sub-division. Finally, we hypothesize that between-group differences in activity patterns are mediated by differences in functional connectivity between regions of the pain matrix and other regions implicated in empathy and the control of self-other representations including but not limited to the rTPJ.

## Methods

### Participants

Forty-four healthy participants (18 males, 26 females) aged between 18 and 42 years (mean = 23.96, S.E = 1.37) volunteered to take part in the study. All participants self-reported being right handed, had normal or corrected vision. Furthermore participants had previously completed the Vicarious Pain Questionnaire (VPQ), an online measure assessing reports and characteristics of conscious vicarious pain experiences (Grice-Jackson et al., 2017). It consists of 16 movies depicting injections (*N* = 8) and sports injuries (*N* = 8). After each movie, participants report whether it triggered pain on your own body (giving a summed score across all movies from 0 to 16). Upon giving an affirmative answer they are then asked follow-up questions namely: to rate the intensity (on a 0–10 scale), to select from a series of pain descriptors that describe sensory and affective qualities of pain (Melzack, 1987), and to indicate whether the pain is localized or generalized. The three groups were derived by performing a two-step cluster analyses on the larger datatset (*n* = 573) that included the fMRI participants (as the method requires a sample size of several hundred). The fMRI sample consisted of 21 non-responder controls, 13 Sensory/Localiser responders and 10 Affective/General responders. The details of the participants, in relation to their performance on the VPQ, is summarized in Table 1. Participants provided written and informed consent in accordance with the Declaration of Helsinki. They were paid £15 for their participation in the study. The study's procedures were reviewed and approved by the Brighton and Sussex Medical School (BSMS) Research Ethics Committee.

**Table 1:** The characteristics of the three groups on the Vicarious Pain Questionnaire (VPQ) showing the mean (SD in parentheses) for the three dimensions used in the cluster analysis, together with mean intensity (0–10 scale).

	**Total pain response**	**Localized-General responses**	**Sensory-Affective responses**	**Average intensity scores**
Controls	0.05 (0.21)	0.05 (0.21)	0.09 (0.43)	0.003 (0.01)
Sensory/Localiser	11.27 (3.06)	3.64 (6.71)	12.63 (6.43)	2.88 (1.77)
Affective/General	11.77 (2.04)	−4.67 (6.53)	−11.44 (10.17)	3.75 (1.46)

*The three dimensions are: the number of movies in which pain is reported (/16); the number of sensory descriptors minus the number of affective descriptors; and the number of pain responses that are localized minus the number of pain responses that are generalized throughout the body*.

### Apparatus

A Siemens Avanto 1.5 Tesla MRI scanner was used to collect all images throughout this experiment. A single row four-button button box was used for tasks 1 and 2 with only the two central buttons active so that participants could indicate movements to the left and right.

### Task materials

This study included a series of 256 images depicting hands and feet experiencing different types of pain that one might experience in the real world (i.e., a fingers being caught in a car door) with contextual matched no-pain images (i.e., a hand closing a car door). The images were taken from a stimuli set provided by Dr Philip Jackson, Université Laval (Quebec) which had been used in a series of fMRI and EEG studies to assess empathy for pain (Jackson et al., 2005; Cheng et al., 2008). This original stimuli set include 128 images all of which showed right hands and right feet. Equivalent images depicting left and right body parts were created by mirror reversal of the images. The images of hands and feet were displayed from a series of orientations with some of the images coming from a position which could be produced by the observer (i.e., the hand/feet coming from the base of the image) and some of which could not be produced by the observer (i.e., the hand of foot comes from the side or top of the image). The visual stimuli were presented on the projector via a stimuli PC using Matlab 2014a and Cogent Toolbox.

### Procedures and design

The stimuli followed a 2 (image condition: pain vs. no pain) × 2 (topography: hand vs. foot) event-related design. Each condition contained 16 trials/image presentations which were randomly drawn from the full image set for each condition. The full session lasted approximately 18 min (~410 volumes).

**Figure 1:**
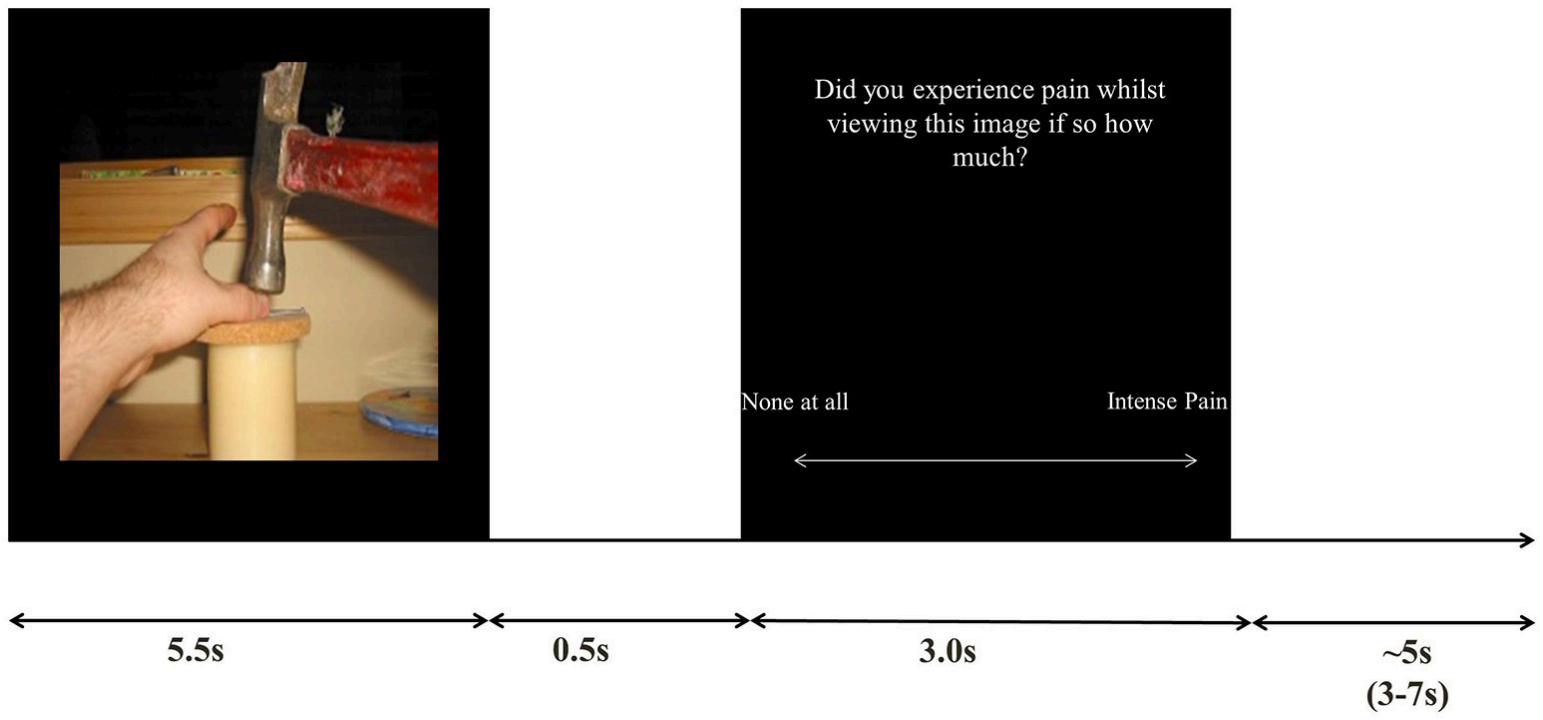
Stimulus example and trial timings.

Trials consisted of viewing an image followed by a judgment as to whether or not they experienced a pain sensation whilst viewing the image, The response was made on a visual analog scale slider which participants controlled with two directions on a button box (left/index finger: no-pain, right/middle finger: intense pain). The image was displayed for 5.5s per trial, followed by a 0.5s blank screen, followed by a 3s pain judgment question (after 3s the response was not taken), followed by a jittered 3-7s inter trial interval (see Figure 1 for trial setup).

### Scanning protocol

Functional fMRI data were collected using an interleaved series sequence with a −30° AC-PC image plane which was fit to include the top of the somatosensory cortex and the temporal poles (TR = 2,620, TE = 43, FOV = 192 × 192 × 122 mm, Voxel Size 3 × 3 × 3 mm, Slice number = 35). In addition to the EPI data collected a structural T1-weighted wide-GRAPPA MPRAGE sequence was acquired for use in coregistration (TR = 2,730 ms, TE = 3.57 ms, FOV = 240 × 256 × 192 mm, voxel size = 1 × 1 × 1 mm). Participants were laid supine on the scanner's bed before being moved into the coil and a T1 Weighted structural image was acquired which was then followed by the fMRI data acquisition and task. Each participant's fMRI session was split between three tasks, of which the main one (always conducted first) is reported here. The other tasks were a cyberball task of social exclusion, and a pain perception task involving the presence/absence of facial expressions. The whole session lasted for approximately 60 mins.

### fMRI pre-processing

Data was processed using Matlab 2014a SPM8 toolbox. Initially all images were reoriented relative to the anterior and posterior commissure. This was followed by correction of movement during the task by realigning all of the images to the first as well as estimation of the movement parameters for each image and then by coregistration of realigned images with T1 weighted structural images. Raw movement parameters were used to exclude participants for excessive movement during the experiment; specifically if a translation regressor exceeded 3 mm and a rotational regressor exceeded 5°. Two controls and one Sensory/Localizer responder were removed because of excessive movement in the scanner. One more control was removed because of an error in recording the trial triggers on the stimuli display PC. This resulted in 18 controls, 12 Sensory/localized and 10 Affective/generalized responders in the final analysis

EPI images were then normalized to a standardized anatomical brain (MNI- Montreal Neurological Institute) using SPM's Dartel normalization function with default parameters and finally a Gaussian smoothing kernel (8 × 8 × 8 mm) was applied to the images to increase signal to noise ratio. Homogeneity of variance was used as a check for the warping of images during pre-processing.

### fMRI analyses

The same statistical threshold was applied to all analyses and consisted of a cluster-level FDR (False Discovery Rate) of *p* < 0.05 and a minimum cluster extent of 40 voxels. Statistical parametric Mapping 8 (SPM8, Wellcome trust Center for Neuroimaging) with the “MarsBar” (Brett et al., 2002) and “anatomy” (Eickhoff et al., 2005) toolboxes was used for the fMRI analysis. Clusters containing several peak maxima (separated by at least 8 mm) are reported for each cluster when appropriate.

Three sets of first and second level design matrices were created for the fMRI analysis. The first focused primarily on the observations of pain and no pain for the three pain groups. A first level design matrix extracted the parameter estimates for all pain and no pain observations (32 trials per condition; Interscan Interval: 2.62s, Microtime Resolution: 16 Microtime onset: 1). At this stage, movement regressors were included in the model as a covariate. Six ridged body movement regressors were included in the first level models throughout the study which included three rotational (roll, pitch, yaw), and three translational regressors (X, Y, Z). For the between group analyses SPM's contrasts manager was used to measure the difference between Pain and No-pain images with the t-contrast function in order to estimate the parameters for Pain > No-pain contrasts in order to assess the size of the difference between condition for each participant. At the second level a full factorial design was used to model data with 2 factors which included: Pain group (3 Levels: Controls, Sensory/Localisers, Affective generals) and Image observations (2 Levels: Pain vs. No Pain). For the between group analysis a One way ANOVA design was used at the second level using the Pain > No Pain parameter estimates with Pain group used as the analytical factor.

The second set of design matrices was used to model parameter estimates for the ROI analysis using 7 regions of the pain matrix and the rTPJ. The coordinates for the anterior cingulate cortex (ACC) and anterior insula were based on the meta-analysis of Lamm et al. (2011) namely: ACC, *x* = 0 *y* = 12 *z* = 45; left Anterior Insula (AI), *x* = −40 *y* = 22 *z* = 0; and right AI, *x* = 39 *y* = 23 *z* = −4 (in MNI space). The location for the rTPJ was based on the mid-point between the anterior and posterior rTPJ subdivisions discussed in Krall et al. (2014) meta-analysis of the rTPJ namely *x* = 54 *y* = −48 *z* = 22 (MNI space). For these regions a 10 mm spherical binary masks was applied. By contrast, the somatosensory cortex was defined anatomically rather than functionally using the masks on SPM's anatomy tool box (Eickhoff et al., 2005) for four somatosensory regions (left SI, right SI, left SII, and right SII). Parameter estimates (Betas) were extracted from these regions using the “MarsBar” tool box (Brett et al., 2002) for Pain > No-pain contrasts. Due to the somatotopic organization of SI a secondary more detailed ROI analysis was carried out. Separate hand and foot SI ROIs were selected using a 10 mm spherical masks around the left and right hand area reported by Bingel et al. (2004) for physical pain stimulation (left MNI: *x* = 39, *y* = −30, *z* = 51; right MNI *x* = 36, *y* = −36, *z* = 48, left and right hand areas were compiled into the same ROI) and the left and right foot area (left MNI: *x* = −9, *y* = −39, *z* = 57; right MNI *x* = 9, *y* = −36, *z* = 66).

The PPI (Psycho-Physiological Interactions) analysis takes seed regions (in our case, standard regions of the pain matrix identified via our whole brain analyses) and uses linear regression to find correlated activity between these seed regions and all other regions of the brain as a function of a psychological variable (in our case, whether a stimulus depicted pain or not). The PPI models were produced using the Generalized PPI Toolbox (McLaren et al., 2012) and included all the same event-related and nuisance regressors as in the original whole-brain GLM. Additionally, the PPI model included one regressor coding the overall BOLD time course of the seed region, and “Pain Image” regressor coding the PPI interaction term between Pain and No-pain image observation. To examine psychophysiological interactions at the group level, we then specified second-level models similar to those used in the whole-brain GLM of BOLD activations. For each seed region separately, estimates of the PPI interaction terms relating to the “Pain Image” events regressors were entered into a 3(Pain group: Controls vs. S/L Responders vs. A/G Responders) × 2 (Pain Image: Pain vs. No Pain) ANOVA model.

## Results

### Vicarious pain ratings

**Figure 2:**
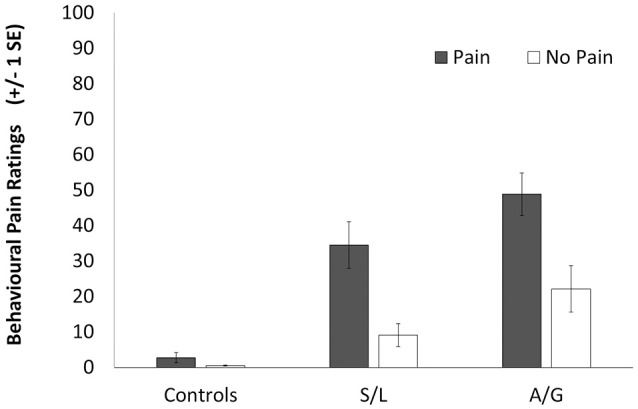
Subjective ratings for the pain (filled bars) and no-pain (empty bars) stimuli. A score of 100 indicates a high perception of pain in the participant in response to the observation of pain and a score of 0 represents no perception of pain. Error bars show ±1 SEM.

The visual analog scale was transformed to a 0–100 range with a higher score indicating higher intensity of vicarious pain. The mean scores, shown in Figure 2, were analyzed using a 2 (Condition: Pain vs. No-pain stimulus) × 3 (Group) ANOVA. There were significant main effects of stimulus [*F*_(1, 39)_ = 68.67, *p* < 0.001, *r* = 0.64] and group [*F*_(2, 39)_ = 34.45, *p* < 0.001, *r* = 0.65] as well as a significant interaction [*F*_(1, 39)_ = 15.554, *p* < 0.001, *r* = 0.47]. Within group planned comparisons showed that the S/L [*t*_(11)_ = 5.217, *p* < 0.001] and A/G groups [*t*_(9)_ = 3.653, *p* = 0.011] showed significantly increased scores during Pain relative to No-pain trials but the controls did not [*t*_(17)_ = 1.655, *p* = 0.155]. In summary, our two responder groups reported increased levels of vicarious pain for these stimuli during scanning as they had previously done for similar stimuli outside the scanner.

### fMRI results

#### Whole brain analysis

**Figure 3:**
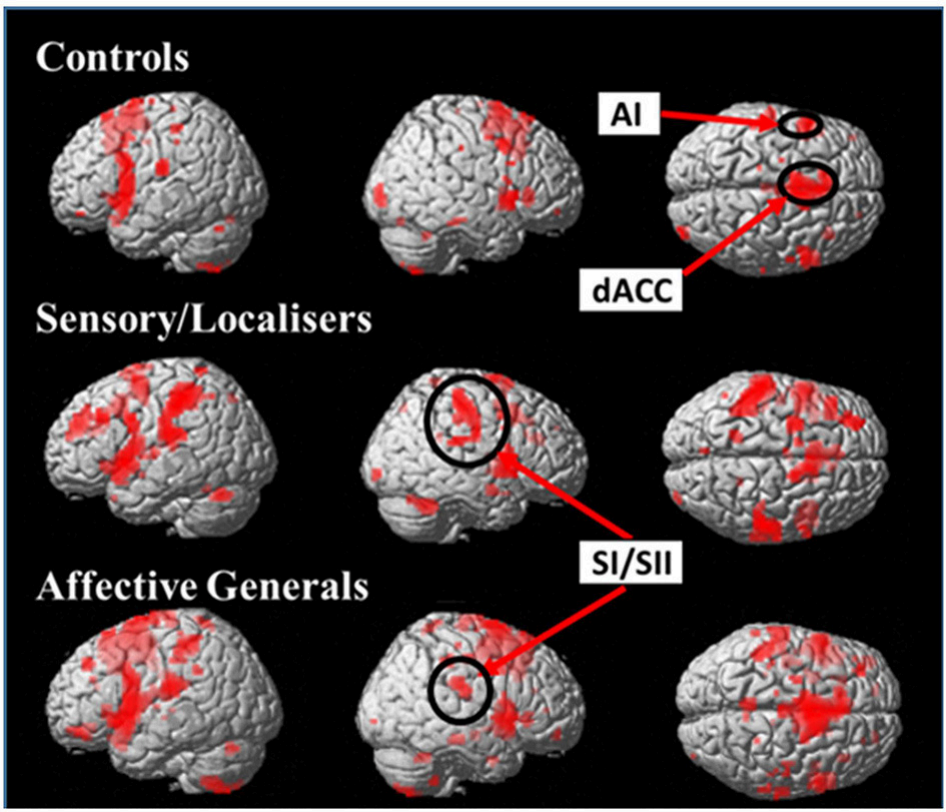
Images display within group effects for Pain > No-Pain contrasts for each of the groups. Contrasts use a whole brain *p* < 0.001 (uncorrected) with a cluster extent threshold of *k* = 10 (the more liberal statistical threshold has only been used for figure display). All groups display increased activation in affective pain matrix regions (dACC + AI) but only S/L and A/G responders show effects in the primary and secondary somatosensory cortices.

Initially a whole brain analysis was run on the data. We assessed pain vs. no-pain within groups and contrasted the effects of the same stimuli between groups. All of the tests were carried out using t-contrasts. Table 2 displays all regions which showed significantly increased differences in pain vs. no-pain activation for the three groups (see also Figure 3). This analysis shows that all groups display effects in regions associated with the affective processing of pain (anterior insula, and dorsal anterior cingulate extending into the supplementary motor area). However, only the S/L and A/G group showed increased activation in the somatosensory cortices which was confirmed by a subsequent ROI analysis.

**Table 2:** Regions showing significant within group effects in Pain > No-pain image contrasts. Peak MNI effects are displayed for each effect as well as FDR corrected cluster significance values.

**Brain Regions**	**Lat**	**MNI Coordinates**			***t*-score**	***k***	**P(FDR)**
		***x***	***Y***	***z***			**Cluster**
**Controls: Pain** > **No pain**							
Supplementary motor area	L/R	0	3	66	4.89	351	<0.001
Anterior insula	L	−54	9	−9	4.62	239	<0.001
Inferior frontal gyrus	L	−57	9	27	4.35		
Anterior insula	R	57	12	−3	4.38	54	0.045
**S/L: Pain** > **No pain**							
Cerebellum (VI)	R	30	−54	−30	5.48	76	0.011
Primary somatosensory cortex (1/2)	L	−54	−30	51	5.20	361	<0.001
Secondary somatosensory cortex	L	−57	−24	21	4.49		
Inferior parietal lobule	L	−50	−33	18	3.92		
Dorsal anterior cingulate cortex	L/R	−3	12	45	5.07	136	0.001
Primary somatosensory cortex (1)	R	57	−27	51	4.76	146	0.001
Primary somatosensory cortex (3b)	R	54	−18	42	3.79		
Anterior insula	L	−33	12	0	4.51	313	0.001
Parahippocampal gyrus	L	−21	5	−21	4.47		
Anterior insula	R	51	3	−3	4.40	105	0.003
Dorsal lateral prefrontal cortex	L	−30	45	27	4.31	113	0.002
Medial prefrontal cortex	L	−20	39	39	3.36		
Precentral gyrus	L	−27	0	60	4.27	64	0.017
Caudate nucleus	R	15	6	6	3.96	69	0.014
**A/G: Pain** > **No-pain**							
Supplementary motor area	L/R	0	3	66	5.42	780	<0.001
Dorsal anterior cingulate cortex	L/R	3	−3	54	4.34		
Inferior frontal gyrus (Opercularis)	L	−57	9	9	4.95	512	<0.001
Temporal pole	L	−48	12	−9	4.67		
Anterior Insula	L	−45	3	3	4.43		
Secondary somatosensory cortex	R	−60	−21	19	4.81	143	0.027
Ventral striatum	R	21	6	−9	4.56	122	0.026
Periaqueductal gray	R	12	6	3	4.42		
Premotor cortex	L	48	−8	56	4.28	137	0.017
Anterior insula	R	42	3	3	4.21	100	0.035
Secondary somatosensory cortex	L	−63	−24	21	4.13	111	0.029

**Figure 4:**
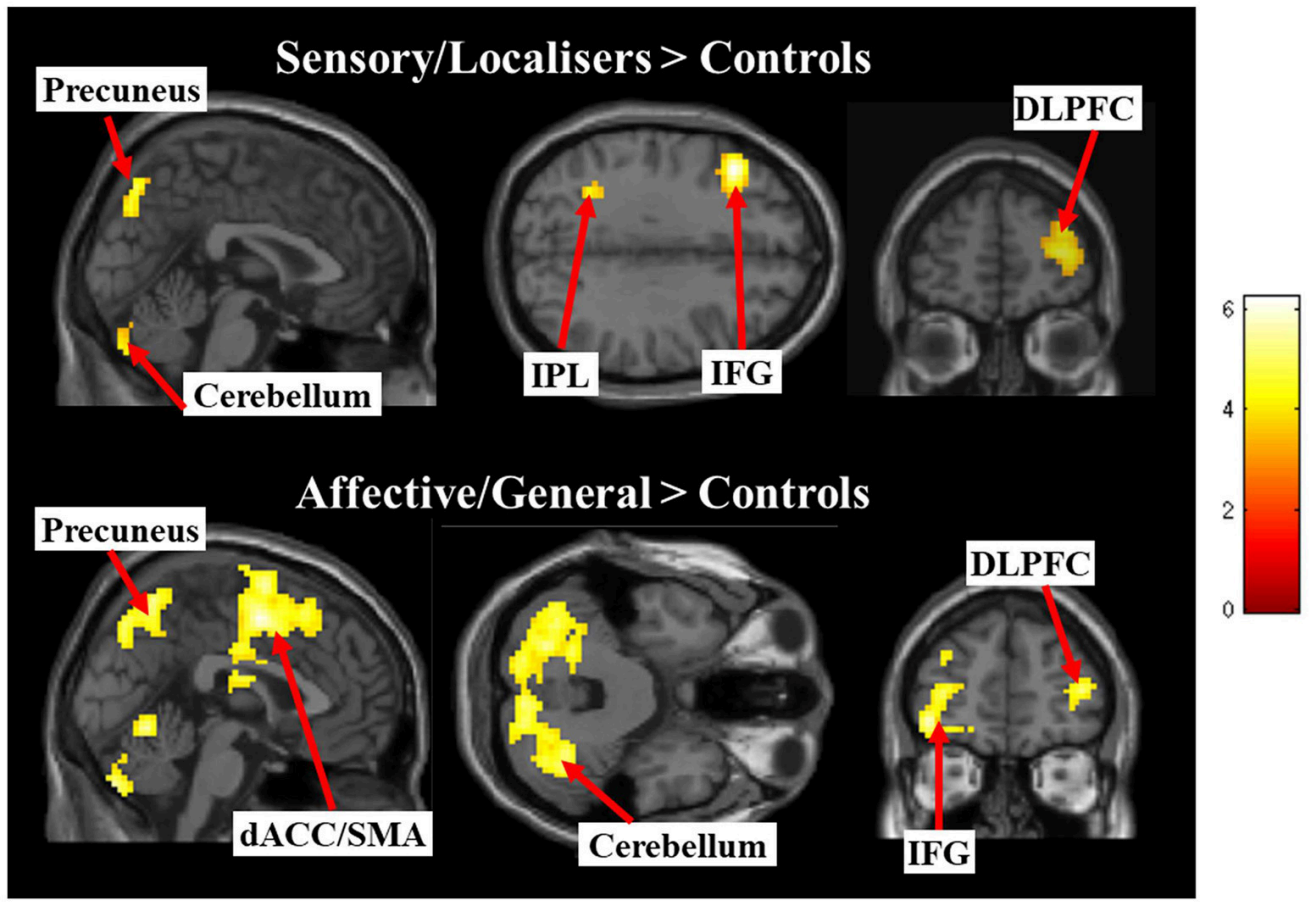
Images display between group effects for Pain > Pain contrasts for each of the responder groups compared with controls. Contrasts use a whole brain *p* < 0.001 (uncorrected) with a cluster extent threshold of *k* = 10 (the more liberal statistical threshold has only been used for figure display).

The differences between groups were explored by assessing Pain vs. No-Pain first level *t*-contrast betas in a second level one way ANOVA with three groups. Figure 4 and Table 3 displays regions showing significant effects (contrasts which did not yield significant effects are not displayed). The responder groups had greater activity than controls in a variety of regions when observing pain, but no effects were observed in the opposite direction (i.e., controls > responders). This included the dorsolateral prefrontal cortex and cerebellum for both sensory/localizer responders and affective/general. No group differences were statistically detected between the two responder groups suggesting they are broadly similar, at least for the present level of statistical power. Notwithstanding this similarity, we demonstrate later that the groups differ in their connectivity profile.

**Table 3:** Regions showing significant between group effects for Pain vs. Pain contrasts in the responder groups relative to contrasts.

**Brain Regions**	**Lat**	**MNI Coordinates**			***t*-score**	***k***	**p(FDR)**
		***x***	***y***	***z***			**Clusters**
**S/L Pain** > **Controls Pain**							
Inferior frontal gyrus	L	−42	24	33	4.03	82	<0.001
Precuneus	L/R	3	−75	51	5.78	251	<0.001
Inferior parietal lobule	L	−36	−54	42	4.69	97	0.005
Dorsomedial Prefrontal cortex	L	−27	51	12	4.82	211	<0.001
Medial Frontal cortex	L	−33	51	24	4.50		
Cerebellum (VI)	R	−33	−57	−30	4.26	56	0.030
Superior frontal gyrus	L	−27	3	63	4.06	41	0.048
Cerebellum (Crus 2)	L/R	−3	−84	−36	4.00	165	<0.001
Cerebellum (Vermis 7)	L/R	0	−75	−24	3.42		
**A/G Pain** > **Controls pain**							
Dorsal anterior cingulate cortex	L/R	2	−5	50	5.21	596	<0.001
Supplementary motor area	L/R	4	−1	51	4.62		
Inferior frontal gyrus	R	53	5	10	5.13	116	0.002
Dorsal lateral prefrontal cortex	L	−45	21	30	5.04	89	<0.001
Primary somatosensory cortex (1)	L	−54	−30	54	4.97	178	0.002
Primary Somatosensory cortex (2)	L	−48	−39	51	3.65		
Cerebellum (Crus 1)	L	−42	−69	−24	4.86	197	0.003
Cerebellum (VII)		−30	−78	−45	4.50		
Ventral premotor area	L	54	2	39	4.81	69	0.016
Anterior insula	L	−50	3	−3	4.74	181	<0.001
Anterior insula	R	48	18	−12	4.47	47	0.050
Precuneus	L/R	−5	−55	51	4.45	144	0.032
Thalamus	R	11	4	2	4.29	108	0.029

*Peak MNI effects are displayed for each effect as well as FDR corrected cluster significance values*.

### ROI analysis

**Figure 5:**
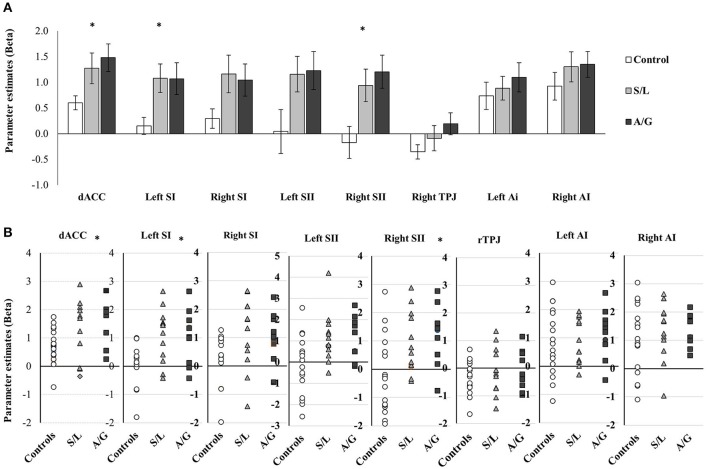
**(A)** Averaged parameter estimate betas extracted from each ROI are displayed for each group. Results of between group ANOVAs are displayed and significant effects are denoted with ^*^p < 0.05. Error bars show ±1 SEM. **(B)** Script plots for showing the individual beta values extracted for the ROI analysis.

Parameter estimates were extracted from each ROI for contrasts between pain vs. no-pain observations. These parameter estimates show the difference between pain and no pain observations with positive beta values indicating increased activation in the region when viewing pain images. A series of one way ANOVAs assessing differences between the pain groups was run on each ROI. Given that these analyses were hypothesis-driven, we considered that a type 2 error would be more detrimental than a type 1 error and therefore opted to report effect sizes (in addition to *p*-values), rather than apply a more conservative Bonferroni correction. Four regions display significant differences between the groups, including: dACC [*F*_(2, 39)_ = 4.714, *p* = 0.015, *r* = 0.635], left SI [*F*_(2, 39)_ = 5.757, *p* = 0.007, *r* = 0.741], and right SII [*F*_(2, 39)_ = 5.441, *p* = 0.021, *r* = 0.704], additionally left SII and right SI showed an effect of borderline significance [Left SII: *F*_(2, 35)_ = 2.846, *p* = 0.076, *r* = 0.589; right SI *F*_(2, 39)_ = 3.114, *p* = 0.056, *r* = 0.491]. For all significant ROIs the two responders groups had significantly higher signal change relative to controls but they did not differ from each other. Non-significant effects included: the right TPJ [*F*_(2, 39)_ = 2.0440.634, *p* = 0.512, *r* = 0.156], the left AI [*F*_(2, 39)_ = 0.431, *p* = 0.653, *r* = 0.097] and the right AI [*F*_(2, 39)_ = 0.761, *p* = 0.474, *r* = 0.150, see Figure 5]. These effects show that both affective and sensory pain matrix regions show differences in activation between the groups with the two pain responder groups displaying increased differences between pain and no pain observations relative to controls. These were driven by differences in the pain rather than no-pain condition.

**Figure 6:**
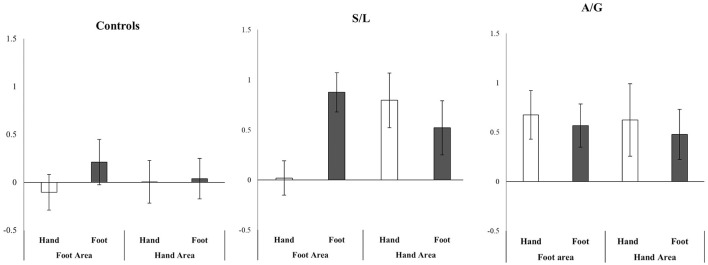
Relative activity in SI hand and foot areas (Pain > No-Pain) depending on whether a hand or foot stimulus was shown. Only the S/L group show an interaction between region X stimulus. Error bars show ±1 SEM.

Due to the somatotopic organization of S1 a more detailed ROI analysis was carried out. The results are summarized in Figure 6. A 2 (hand vs. foot image) × 2 (hand vs. foot ROI) repeated measures ANOVA was run on the data (one for each Group: Controls, S/L, A/G). The S/L group showed a strong interaction of image type X ROI [*F*_(1, 11)_ = 20.40, *p* = 0.001, *r* = 0.63] such that feet images more strongly activated the foot area and hand images more strongly activated the hand area, This pattern was absent in the other two groups [Controls: *F*_(2, 17)_ = 0.356, *p* = 0.559, *r* = 0.021; A/G: *F*_(1, 9)_ = 0.01, *p* = 0.95, *r* = 0.001]. No group showed main effects of image type [Controls: *F*_(1, 17)_ = 0.07, *p* = 0.79, *r* = 0.06; A/G: *F*_(1, 9)_ = 0.39, *p* = 0.55, *r* = 0.04; S/L: *F*_(1, 11)_ = 1.65, *p* = 0.22, *r* = 0.12] or ROI [Controls: *F*_(2, 17)_ = 0.48, *p* = 0.50, *r* = 0.03; A/G: *F*_(1, 9)_ = 0.05, *p* = 0.83, *r* = 0.01; S/L: *F*_(1, 11)_ = 1.00, *p* = 0.34, *r* = 0.08]. However, it is to be noted that the triple interaction, if treated in a single ANOVA (group X image type X ROI), was not significant hence this result should be regarded as preliminary and in need of further replication.

### Functional connectivity analysis

We specified 5 psychophysiological interaction models to test our hypothesis that regions associated with mirror pain activation in the responder group would show differential patterns of functional connectivity depending on whether or not they viewed pain or not. Each of the 5 models involved a different seed region that had been identified as showing a significant “pain > no pain” main effect in the GLM of BOLD activations. These regions included: the left SI/SII ([−57, −24, 21], *k* = 641), the left AI ([−51, 6, 09], *k* = 802), the right AI ([54, 12, −3], *k* = 475), the dACC/SMA ([−3, 18, 42], *k* = 641), and the right SI/SII ([54, −21, 42], *k* = 390). Each seed region was entered into first level models to estimate connectivity between the seeds and other regions during the session. The five PPI analyses were investigated by comparing pain vs. no-pain images for each group, and between group contrasts, comparing pain and no pain images across the groups. All contrasts which showed significant PPI effects are displayed in Table 4 which includes directions of effects and the location of significant effects.

**Table 4:** Regions showing significant PPI effects for within group contrasts for at the whole group level and for each pain responder group.

**PPI seed**	**Brain regions**	**Lat**	**MNI Coordinates**			***t*-score**	***K***	**p(FDR)**
			***x***	***y***	***z***			
**S/L Pain** >**No-Pain**								
Left SI/SII	Angular gyrus (IPL – PGp)	L	−39	−75	42	4.77	58	0.050
Right SI/SII	Angular gyrus (IPL – PGa)	L	−36	−75	57	4.31	119	0.002
	Angular gyrus (IPL – PGp)	L	−45	−72	33	3.93		
Left AI	Retrosplenial cortex	R	9	−60	42	5.02	62	0.028
Left AI	Temporo-parietal junction	R	36	−51	36	5.18	62	0.028
	Intraparietal sulcus	R	42	−51	45	4.06		
Right AI	Temporo-parietal junction	R	42	−54	45	4.36	66	0.024
dACC/SMA	Angular gyrus (IPL - PGp)	L	−39	−75	42	4.48	218	<0.001
dACC/SMA	Middle frontal gyrus	L	−39	21	48	4.52	106	0.001
	Anterior prefrontal cortex	L	−30	9	50	4.25		
	DLPFC	L	−48	18	39	4.25		
dACC/SMA	DLPFC	R	51	30	21	5.53	55	0.021
**A/G Pain** > **No-pain**								
Left SI/SII	DMPFC	L	−9	54	15	4.85	78	0.044
	Medial frontopolar cortex	L	−12	51	5	4.14		
Left AI	DLPFC	L	−42	48	−3	4.64	91	0.003
**S/L (Pain** − **No-Pain)** > **Control (Pain** − **No-Pain)**								
ACC	DLPFC	R	51	30	21	5.32	44	0.021
Left AI	Temporo-parietal junction	R	42	−51	45	4.89	91	0.003
	Intraparietal sulcus	R	35	−51	35	4.81		
Right AI	Temporo-parietal junction	R	42	−51	48	4.65	60	0.034
	Intraparietal sulcus	R	42	−45	39	3.50		

*Effects are displayed for each seed region's PPI analyses and the direction of the effects are displayed. Only contrasts showing significant effects are displayed*.

For the S/L group, the left and right anterior insula seed regions showed greater functional connectivity (contrasting Pain > No-pain) with the rTPJ. It is to be noted that although we initially hypothesized a role of the rTPJ, this finding emerged from a whole-brain data-driven approach. Seed regions in other parts of the pain matrix (left and right SI/SII and dACC/SMA) showed greater functional connectivity (Pain > No-pain) with a region in the left posterior angular gyrus. There were three other PPIs observed in the S/L group (involving retrosplenial cortex and bilateral prefrontal regions). By contrast, for the A/G group there were only two observed PPIs when contrasting Pain > No-pain (between the left SI/SII seed and dorsal mPFC and left anterior insula seed and left DLPFC). Control groups did not show any significant PPI effects throughout the analysis.

## General discussion

The aim of our study was to assess empathy for pain from the unique perspective of individual differences in self-reported vicarious pain experiences. Previous research has been largely driven from the assumption that seeing someone in pain leads to an implicit simulation of pain, but not to reportable experiences of pain. This assumption has been criticized on the basis of being a reverse inference—just because the pain matrix is active we can't conclude that it corresponds to pain *per se* because these brain regions are also activated in certain other non-pain contexts too (Iannetti et al., 2013). Our own research, and that of others (Osborn and Derbyshire, 2010; Fitzgibbon et al., 2012b), suggests that a significant proportion of people (a quarter to a third) do experience reportable pain-like experiences form observing others in pain. For these individuals, at least, there is less concern about reverse inference (because pain is reported by the participant rather than inferred by the experimenter) and it is possible to explore whether the “standard” findings from the empathy-for-pain literature are driven by this significant minority of participants or do indeed reflect a normative (i.e., universal) response.

Our approach was to take a commonly used paradigm and stimulus set from the literature (hands and feet in painful and non-painful scenarios) and re-examine it from the perspective of differences between “responders” who reliably report the pain of others, and “non-responders,” who do not. Our recent research has shown that responders can be classified in two ways, Sensory/Localized and Affective/General, and these were contrasted against non-responder controls. We hypothesized that these groupings may differentially activate regions of the pain matrix that have been labeled as affective/motivational (e.g., anterior insula, mid-cingulate) and sensory/discriminative (e.g., somatosensory cortices). This was not found. In terms of their pattern of activity, the two responder groups were similar to each other but were different to the controls. All three groups tended to activate the anterior insula and mid-cingulate regions (extending into supplementary motor area) when contrasting pain against no-pain. These regions are involved in the awareness of bodily and affective states including but not limited to pain (e.g., Gu et al., 2013). The two responder groups also tended to activate primary and secondary somatosensory cortices when observing pain, which was not found for the controls. There was preliminary evidence that the Sensory/Localized group did so in a more somatotopic manner (e.g., feet in pain activating the foot area) whereas the Affective/General group did so in a more whole-body manner. In normative samples (i.e., that do not separate out responders who report pain), images such as these have been reported to activate the somatosensory cortices (Lamm et al., 2011). As such, our findings raise the possibility that these previous results in somatosensory cortex are driven mainly (perhaps exclusively) by a subset of the “normal” population who are having pain-like experiences rather than reflecting a normative tendency to implicitly simulate the sensory properties of pain. More recent imaging research that uses a pattern analysis approach has argued that vicarious pain of different body parts does not involve somatosensory regions (Krishnan et al., 2016), and it would be important to use this approach on the individual differences we have observed. However, the use of somatosensory regions to support vicarious pain in sensory/localized responders is consistent with our own previous research suggesting that EEG mu/beta suppression is found only in this group (Grice-Jackson et al., 2017). It is also consistent with the only other previous fMRI (Osborn and Derbyshire, 2010) and EEG (Fitzgibbon et al., 2012a) studies of mirror-pain responders which also show greater recruitment of somatosensory regions by these groups. Although there are commonalities amongst all three groups in terms of pattern of activity, and further commonalities between the two responder groups, the analysis of functional connectivity revealed dissociable patterns. For the control group, we found no significant effects. For the responder groups, there were ten significant effects (all in frontal and parietal regions) and primarily for the Sensory/Localized group. Importantly, many of the same regions emerged via multiple independent analyses (i.e., using different seed regions).

Before discussing these findings in more detail it is important to acknowledge the limitations of the current study. Firstly, the study is relatively under-powered and this may explain why the whole-brain results did not yield group X pain/no-pain interactions. However, group X pain/no-pain interactions were evident in the PPI analysis, the behavioral ratings, and the ROI analyses. Secondly, future research should consider more sophisticated analyses that move away from the notion of a generic “pain matrix” and instead use voxel-based subject-specific classifiers that are sensitive to the presence of physical pain. Thirdly, the relationship between these three groupings of vicarious pain needs to be understood in more detail. Here we assumed that there are three independent groups, but alternative scenarios are possible such as hierarchical nesting within a multi-level model (e.g., with responder/non-responder at the first level, and the “responder” group subdivided into S/L and A/G subtypes). Finally, the generality of the findings to other stimuli and scenarios is unclear. In the present study, participants attended to their own vicarious pain response. It would be important to assess whether the results change if participants have to rate the pain intensity felt by the other person (i.e., adopting a third-person perspective).

Having considered limitations, we shall discuss the potential role of the parietal regions (rTPJ, left angular gyrus, precuneus/retrosplenial) and frontal regions (DLPFC, mPFC) in vicarious pain. One region that was hypothesized from the outset to be important was the rTPJ and this region showed greater connectivity to the left and right anterior insula in the S/L group when observing pain (relative to no-pain; and also relative to the same contrast in controls). One function of this region is linked to acting as a switch between self and other based representations such that increased rTPJ activity is linked specifically to suppressing the dominant self-perspective and enhancing the other perspective (Bird and Viding, 2014), as well as having a wider role in attentional control (Wu et al., 2015). One study contrasted physically painful stimuli presented concurrently with images of other people in painful or neutral situations (Godinho et al., 2012). Seeing someone else in pain increases the self-reported intensity of physical pain (a normal form of self-other confusion) and this was linked to the same posterior region of the rTPJ that we observed (and not to increased activity in the pain matrix). The pattern analysis study of Krishnan et al. (2016) found that the rTPJ (and other regions of the mentalizing network, but not the pain matrix) were involved in vicarious pain, although in this study participants performed perspective taking (imagining someone else's experiences on their own body). Our explanation of the Sensory/Localized group is that they systematically fail to attribute shared bodily representations to others and this, at least in part, reflects structural and functional differences within the rTPJ coupled with other differences (e.g., in left parietal cortex).

Aside from the rTPJ, other regions were highlighted by the connectivity analysis for the S/L group. A region in the left angular gyrus showed greater connectivity (when observing Pain > No-pain) to three seed regions (left and right somatosensory cortices, and the cingulate/SMA region). This parietal region is not the left hemispheric homolog of the rTPJ but is several centimeters posterior to it. This region has been found to be important in several studies relating to agency and body ownership. Long-term gamers who habitually use a certain avatar activate this region when thinking about (Ganesh et al., 2012) or observing (Lemenager et al., 2014) their avatar. Patients with left parietal lobe damage are more inclined to claim agency to third-person perspective hand movements executed by others (Sirigu et al., 1999), and TMS over this region in healthy controls disrupts agency attribution (Chambon et al., 2015). The precise function(s) of this region is uncertain, but one theory is that it computes a mental simulation of the self into alternate spatial scenes and perspectives (Buckner and Carroll, 2007). The precuneus/retrosplenial area was also implicated by multiple independent analyses (Pain > No-pain in both S/L and A/G groups, and PPI connectivity to left anterior insula in the S/L group). While this region may also serve a general role in mental simulation/imagery (Cavanna and Trimble, 2006), it has also been hypothesized to have a more specific role in pain. Stimulation of this region in rats has an analgesic effect (Rossaneis et al., 2011) and, in humans, patients with fibromyalgia have higher resting levels of activity in this region which may arguably reflect an analgesic function (Wik et al., 2003).

With regards to the frontal lobe, two regions (medial PFC and dorso-lateral PFC) are noteworthy. The medial PFC region was implicated in the functional connectivity analysis for the Affective/General group. It has been linked specifically to the self-concept (e.g., thinking about one's own characteristics) rather than bodily self (e.g., Mitchell et al., 2005). This particular result needs to be treated with caution given that the region was not implicated by any other analysis. The DLPFC has widespread effects on cognitive control (Duncan, 2010) including empathy (Moriguchi et al., 2007) and emotion regulation (Ochsner et al., 2012), so it would be unwise to infer a specific role for mirror pain. The region (both left and right) was implicated across multiple analyses. It is important to explore the role of this region, alongside the parietal regions previously discussed, using methods such as non-invasive brain stimulation (NIBS) and combined NIBS-fMRI to examine its causal role on vicarious pain perception.

To conclude, our research has important theoretical implications for research on empathy for pain (and other shared states). It suggests that greater attention should be paid to individual differences in reportable experiences. These have the potential to distort what is assumed to be a normative response. In particular, activity within the somatosensory cortex when observing others in pain may be primarily (and possibly exclusively) linked to those individuals who report pain when seeing others in pain. Patients with congenital pain insensitivity activate some regions of the “pain matrix,” notably insula and mid-cingulate, when viewing others in pain but notably not the somatosensory cortex (Danziger et al., 2009). Contrary to our initial predictions, the amount of activity in somatosensory cortices does not seem to strongly reflect the distinction between Sensory/Localized and Affective/General, but they may nonetheless show differences in how these regions are activated (body-part vs. whole body respectively).

Increased activity within pain matrix regions might be proposed to be both necessary and sufficient for consciously experienced vicarious pain. We previously referred to this as Threshold Theory (Ward and Banissy, 2015) and is based on the notion that all individuals activate, to varying degrees, the pain matrix on seeing pain but only those that do so above a threshold for awareness have reportable pain-like experiences. We do not doubt that this is part of the explanation, however, we question whether it is sufficient. In particular, we argue that it is interactions between the pain matrix and various fronto-parietal regions that give rise to these reportable vicarious pain experiences. This is more clearly the case for the Sensory/Localized group for whom we observed enhanced functional connectivity between pain matrix regions and the rTPJ and left angular gyrus, both of which are implicated in discriminating self from other and bodily perspective taking. The explanation for the Affective/General group is presently lacking and, in some respects, appears to be intermediate between the Sensory/Localized and the non-responder groups. One possibility is that this group reflects differences on autonomic measures (for a model incorporating this see Giummarra and Fitzgibbon, 2015). In summary, our research provides fresh evidence that these individual differences are important to consider methodologically (as they can skew results) and theoretically, as they provide important test cases for current models.

## Author contributions

TG collected and analyzed the data. TG, MB, HC, and JW all contributed to the design of the study, interpretation of data, and writing of the manuscript.

### Conflict of interest statement

The authors declare that the research was conducted in the absence of any commercial or financial relationships that could be construed as a potential conflict of interest.
